# Maternal Immune Activation Leads to Mitochondrial Dysfunction and a Social Deficit in Offspring That Is Reversed by Nicotinamide Riboside

**DOI:** 10.3390/nu18060889

**Published:** 2026-03-11

**Authors:** Arkadiy A. Bazhin, Ekaterina S. Solodnikova, Daniel A. San Miguel, Robert Dantzer, Yezaz A. Ghouri, Jennifer J. Donegan, Elena Goun

**Affiliations:** 1 Department of Chemistry, University of Missouri, Columbia, MO 65211, USA; abvmh@missouri.edu (A.A.B.); esphk@missouri.edu (E.S.S.); 2 College of Pharmacy, Division of Pharmacology & Toxicology, The University of Texas at Austin, Austin, TX 78712, USA; dsanmiguel@utexas.edu; 3 Department of Symptom Research, The University of Texas MD Anderson Cancer Center, Houston, TX 77030, USA; rdantzer@mdanderson.org; 4 Division of Gastroenterology & Hepatology, St. Louis University, St. Louis, MO 63110, USA; yezaz.ghouri@ssmhealth.com; 5 Department of Psychiatry and Behavioral Sciences, Dell Medical School, University of Texas at Austin, Austin, TX 78712, USA

**Keywords:** maternal immune activation, mitochondria, social interaction, prefrontal cortex, nicotinamide riboside

## Abstract

**Background:** Maternal immune activation (MIA) during pregnancy is a known risk factor for several neurodevelopmental and psychiatric disorders, including schizophrenia. In rodent models, MIA is commonly induced using polyinosinic/polycytidylic acid (Poly(I/C)), a viral mimetic that activates Toll-like receptor 3 (TLR3) signaling and elicits an inflammatory response in both the dam and the fetuses. MIA results in various behavioral abnormalities in offspring, including deficits in social interaction. Recent studies have shown that MIA decreases the ability to maintain mitochondrial membrane potential (ΔΨm), the electrical component of the electrochemical gradient required for ATP production and alters mitochondrial protein expression in brain tissue isolated from adult offspring. **Methods:** In the present study, we monitor ΔΨm non-invasively in vivo using a previously published bioluminescence probe in juvenile and adult MIA offspring. We then investigated gene expression in the medial prefrontal cortex of MIA offspring by analyzing a previously published RNA sequencing dataset in combination with MitoCarta3.0, a comprehensive inventory of genes involved in mitochondrial function. Finally, we tested the hypothesis that this mitochondrial dysfunction contributes to the behavioral deficits observed in MIA offspring. **Results:** We have observed impaired ΔΨm maintenance in juvenile MIA offspring that persists into adulthood. Also, we found that MIA alters the expression of many genes associated with mitochondrial energy production. We demonstrated that nicotinamide riboside, a precursor to NAD^+^ known to restore ΔΨm, significantly attenuates MIA-induced social interaction deficits. **Conclusions:** Together, these findings highlight mitochondrial function as a promising therapeutic target for symptoms associated with schizophrenia and support the potential for drug discovery aimed at enhancing mitochondrial health.

## 1. Introduction

While the prevalence of schizophrenia is less than 1 percent, this disorder is a leading cause of disability and premature death worldwide [1,2,3,4,5,6,7]. The etiology of schizophrenia involves a complex interplay between genetic and environmental factors, and substantial epidemiological evidence suggests that maternal infection and the resulting immune response during pregnancy are associated with an increased risk of developing schizophrenia in the offspring [8,9]. For example, prenatal influenza infection during the second trimester leads to an eight-fold increase in risk of offspring developing schizophrenia [10,11] To examine the effect of maternal immune activation (MIA) on the development of neural circuits and behaviors associated with schizophrenia, we used the viral mimetic, polyinosinic/polycytidylic acid (Poly(I/C)). Poly(I/C) is a piece of double-stranded RNA, which activates toll-like receptor 3 (TLR3) and increases the expression of proinflammatory cytokines (IL-1β, TNF-α, IL-6) and type I interferons (IFNα and IFNβ) in both the dam and the fetal brain [12]. MIA offspring show characteristic behavioral deficits, including impairments in complex social behaviors, which emerge in late adolescence or adulthood [13,14,15,16,17,18].

Recently, mitochondrial dysfunction has received attention for its potential role in the pathology of schizophrenia [19,20,21,22]. In genome-wide association studies, investigations of *de novo* mutations, and transcriptome and proteome analyses, more than 50 genes encoding mitochondrial proteins have been implicated in schizophrenia [19]. Post-mortem studies demonstrate reductions in total mitochondria number, mitochondrial protein expression, and respiratory chain enzyme function in the brains of schizophrenia patients [23,24,25,26]. More recently, human-induced pluripotent stem cells derived from people with schizophrenia demonstrated alterations in mitochondrial structure and function [27,28,29,30]. Although mitochondria serve multiple cellular functions, their primary role is to provide energy in the form of ATP through oxidative phosphorylation (OXPHOS). The electrochemical gradient across the inner mitochondrial membrane, the mitochondrial membrane potential (ΔΨm), drives ATP synthesis. Changes in ΔΨm can signal metabolic stress within a cell, and ΔΨm is disrupted in nearly all disorders associated with mitochondrial dysfunction, including diabetes, cardiovascular disease, and neurodegeneration [31,32,33,34]. For these reasons, ΔΨm is often used as a proxy for mitochondrial function [31]. The ability to maintain ΔΨm, particularly during periods of stress and high ATP demand, is essential for normal neurodevelopment and synaptic plasticity in adulthood [35,36]. Multiple lines of evidence suggest a strong interconnection between mitochondrial function and immune signaling [37]. Emerging evidence from recent studies highlights a connection between maternal inflammation during pregnancy and altered mitochondrial function in the offspring’s brain [29,38,39,40]. For instance, MIA has been shown to alter the expression and activity of electron transport chain proteins in adult offspring [41,42,43,44]. In addition, two studies demonstrated that MIA reduces ΔΨm in ex vivo neurons derived from MIA-exposed offspring [29,38]. In the current experiments, we used a novel mitochondria-activatable luciferin (MAL3) for non-invasive, longitudinal monitoring of ΔΨm. Specifically, we used the MAL3 probe to monitor ΔΨm in vivo in juvenile and adult MIA offspring [45]. We demonstrated that MIA impairs the ability to maintain ΔΨm in the developing brain, and that this deficit persists into adulthood. To our knowledge, this is the first in vivo non-invasive demonstration of MIA-induced mitochondrial dysfunction. Further, we showed that MIA alters the expression of genes associated with mitochondrial energy production, and our results are in agreement with recently reported data describing downregulation of other genes involved in mitochondrial dysfunction [40].

In addition, we examined the therapeutic potential of nicotinamide riboside (NR), a form of vitamin B3 and an NAD^+^ precursor marketed as an anti-aging supplement. We previously demonstrated that NR restores ΔΨm in aged animals [45]. In the present study, we demonstrated for the first time that NR supplementation attenuates MIA-induced deficits in social interaction.

Collectively, our findings suggest that MIA induces mitochondrial dysfunction that begins in early neurodevelopment and persists into adulthood. Moreover, our results show that targeting this dysfunction can mitigate behavioral deficits, supporting mitochondrial health as a promising therapeutic target for symptoms associated with disorders such as schizophrenia. These findings also point to a new direction for drug discovery—developing compounds that enhance mitochondrial function.

## 2. Materials and Methods

**Animals**. All experiments were performed in FVB-luc^+/+^ (FVB-Tg(CAG-luc,-GFP)L2G85Chco/J) mice. Animals were initially purchased from Jackson Laboratory (Strain #008450; Bar Harbor, ME, USA) and bred in-house at the Dalton Cardiovascular Research Center (AAALAC-accredited) at the University of Missouri-Columbia. Animals were housed under standard conditions at the same facility in accordance with institutional guidelines: 12 h light/12 h dark cycle, 20–23.3 °C, 30–70% humidity, and given free access to food and water. All interventions were approved by the University of Missouri Institutional Animal Care and Use Committee (protocol# 33501, approved on 10 January 2022). All methods were performed in accordance with the relevant guidelines and regulations.

**Materials**. High molecular weight (average MW 1.5–8 kb) Poly(I/C) was purchased from Invivogen (catalog #31852-29-6; San Diego, CA, USA). Fatty acid-free bovine serum albumin (BSA) and 2,4-dinitrophenol (DNP) were purchased from Millipore-Sigma (Burlington, MA, USA). Saline was received from Intermountain Life Sciences (West Jordan, UT, USA). Both components of the MAL3 probe (TPP-CL2 and azido-TPP1) were synthesized in-house, as described previously [45]. Phosphate-buffered saline (PBS) and dimethylsulfoxide (DMSO) were purchased from Gibco (Thermo Fischer Scientific, Waltham, MA, USA). NR chloride was bought from Xi’an Pincredit Bio-tech Co., Ltd (Xi’an, China).

**Maternal immune activation (MIA) model**. To induce maternal immune activation, Poly(I/C) was used as previously described [46,47]. Briefly, Poly(I/C) was dissolved in physiological water (0.9% NaCl, provided by the manufacturer), heated to 65–70 °C for 10 min. After cooling, the Poly(I/C) was aliquoted and stored at −20 °C. For the timed mating procedure, one male and one female mouse were placed together in a cage in the evening. The next morning, gestational day (GD) 0.5, the male mouse was removed from the cage. On GD 12.5, the pregnant female received an intraperitoneal (i.p.) injection of Poly(I/C) solution in physiological saline (10 mg/kg). Control animals received a vehicle injection at the same time point. Pups were weaned at 21 days postpartum and housed in groups by sex. All experiments included male and female pups from different litters—8 litters for the control group and 8 litters for the Poly(I/C) group.

**Mitochondrial membrane potential (ΔΨm) monitoring**. To determine whether MIA alters mitochondrial function, ΔΨm was measured longitudinally in vivo, using the MAL3 probe as previously described [45]. Two groups were compared: Control (saline-treated, n = 30) and MIA (Poly(I/C)-treated, n = 29). Number n represents the number of mice (single animal) of both sexes. The total number of mice per experiment was 59. The sample size was determined based on our previous experience and G*Power calculations (v 3.1.9.6, Heinrich Heine University, Düsseldorf, Germany). The previous experience allowed us to evaluate the effect size (5.0 − 4.7 = 0.3) and the standard deviation (0.31) for normalized luminescence. Mice were allocated to the control and MIA group based on the treatment they received during the early development stage (GD 12.5). The numbers of males and females were approximately equal in both groups. No other randomization was performed for the allocation. No confounders were controlled. No blinding was performed.

**Figure 1:**
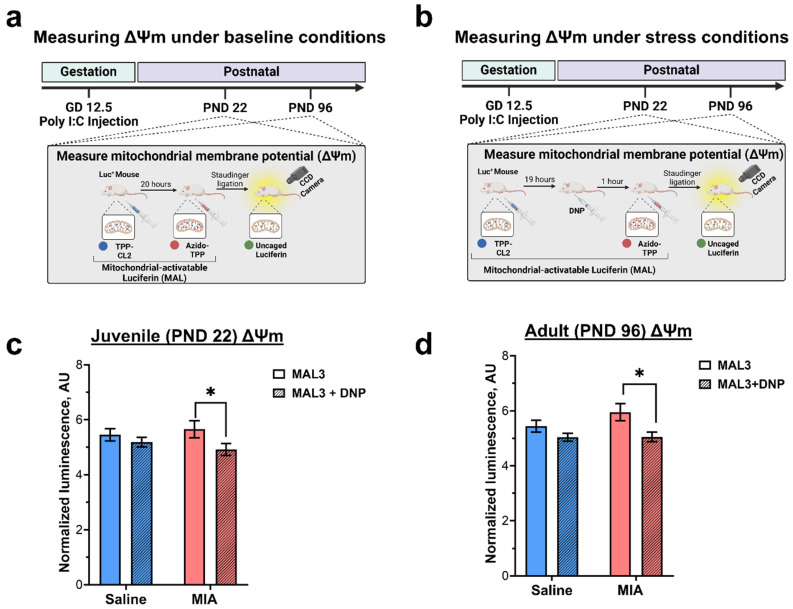
MIA produces persistent deficits in mitochondrial function in vivo. The mitochondria-activatable luciferin (MAL3) [45] probe was used to measure ΔΨm in a non-invasive longitudinal manner. The MAL3 probe consists of two components that were administered as previously reported (Azido-TPP1 and TPP-CL2), which result in the release of free luciferin proportional to ΔΨm and the subsequent bioluminescent signal (**a**,**b**) [45]. The measurements were performed in control and MIA offspring under baseline conditions (**a**) (Created in BioRender. Donegan, J. (2026) https://BioRender.com/rbgldnt) and after a DNP (1 mg/kg) challenge (**b**) (Created in BioRender. Donegan, J. (2026) https://BioRender.com/s85csyl). (**c**) The ΔΨm-specific signal in juvenile animals (PND 22) at baseline (MAL3) and under stress (MAL3 + DNP) conditions. (**d**) The ΔΨm-specific signal in adult animals (PND 96) at baseline (MAL3) and under stress (MAL3 + DNP) conditions. Data were collected from the entire bodies of the animals. Results are presented as mean ± SEM. *p* values were calculated by two-way ANOVA; Fisher’s LSD test was used for the post hoc analysis. *—*p* < 0.05. In the juvenile period, n = 30 for the saline group and n 29 for MIA. In adulthood, n = 22 in the saline group and n = 25 in the MIA group.

Briefly, on postnatal day (PND) 22 or PND 96 (Figure 1a), MIA offspring first received an intravenous (i.v.) injection of TPP-CL2 (100 µL of 1 mM solution in 0.1% BSA in PBS). Twenty hours later, the baseline bioluminescent signal from each mouse was quantified by IVIS Lumina X5 (PerkinElmer, Waltham, MA, USA) every 3 min for 15 min (180 s exposure, binning 16, F1). Then, each mouse received an i.p. injection of 100 µL of a 10 mM solution of azido-TPP1 in PBS. Immediately after that, mice were returned to IVIS Lumina X5 and imaged for 60 min using the same settings. The resulting data were analyzed using the Living Image software (v.4.8.2, PerkinElmer, Waltham, MA, USA), with the entire body as the region of interest (ROI). The bioluminescence signal was integrated over time separately for the period after TPP-CL2 administration alone and for the period after the subsequent injection of azido-TPP1. The integrated signal from TPP-CL2 alone was used for signal normalization. The normalized luminescent signal was plotted as a bar graph using GraphPad Prism v9.3.1 (Boston, MA, USA). No animals were excluded from the analysis, and no criteria were used for excluding or including animals from the analysis.

To determine ΔΨm under conditions of mitochondrial stress, the mitochondrial uncoupler, DNP, was used (Figure 1b). In juvenile and adult mice, 1 week after the initial experiment, DNP (1 mg/kg, prepared in a DMSO/water (1:1) mixture) was administered 19 h after the TPP-CL1 injection. Then, ΔΨm measurements were performed as described above. No animals were excluded from the analysis, and no criteria were used for excluding or including animals from the analysis. The normalized luminescence signal was used as the only outcome measure in this experiment.

Differential expression of bulk RNA-Seq data. To determine whether MIA alters the expression of genes associated with mitochondrial function, we analyzed data from a previous study [43]. Data were obtained from the Gene Expression Omnibus under the GEO Series accession GSE150481. The NextFlow 3.14 nf-core/rnaseq pipeline [48] was used to analyze the RNA-seq data. Reads were aligned to the GENCODE GRCm39 reference genome using STAR [49], and RSEM [50] was used to quantify gene expression. Differentially expressed genes (DEGs) were identified using DESeq2 [51]. PCA from the DESeq2 package revealed an extreme outlier, which was subsequently omitted from further analyses [52]. For the remaining samples (2 controls and 6 MIA), a log2 fold change cutoff of 1.1 was used to better capture genes differentially expressed, given the relatively small fold changes observed across the dataset. Differential expression was interpreted using shrinkage-estimated log2 fold changes rather than applying a large post hoc fold-change cutoff. The DESeq2 framework emphasizes the estimation of stable effect sizes and recommends evaluating biological relevance within the statistical model instead of filtering genes after testing, because the number of detected genes depends strongly on sample size and dispersion characteristics rather than on a universal magnitude threshold [51]. To improve the reliability of effect-size estimates in this low-replicate dataset, log2 fold changes were regularized using the apeglm shrinkage method [53], which reduces variance in fold-change estimates when information is limited and yields more reproducible estimates of transcriptional differences. Significance was defined by an adjusted *p*-value of 0.05. The volcano plot was generated using the R package EnhancedVolcano [53], and the heatmap was created using pheatmap. The analysis included only protein-coding genes, and low-expressing genes were filtered out, retaining only those with a count of at least 5 in 50% or more of the samples. We restricted our analysis to mitochondrial genes only using the MitoCarta3.0 dataset [54], which provides an inventory of 1140 mouse genes that encode proteins localized to mitochondria, with sub-mitochondrial compartment and pathway annotations.

NR-enriched diet. NR can increase cellular energy metabolism, and we previously showed that 2 weeks of 400 mg/kg/day NR administration in aged mice increased ΔΨm [45]. Therefore, NR was administered through the diet to determine whether increasing ΔΨm attenuates behavioral deficits in MIA offspring. The enrichment of standard chow, 5053—Purina PicoLab Rodent Diet 20 (LabDiet, PMI Nutrition International, Richmond, IN, USA), with NR chloride (NR-Cl) was performed in-house as described previously [45]. Briefly, the chow pellets were milled into a powder. Then, the NR-Cl solution in ultrapure water (18 MΩ/cm) was added to the powder to achieve a 0.24% NR concentration in the diet, corresponding to an approx. 400 mg/kg dose per day. The resulting paste was thoroughly mixed to evenly distribute NR, and pellets were formed. Once dried, the pellets were stored at −80 °C. On PND143, MIA offspring were divided into two groups. One group remained on the standard chow diet, while another group received the NR-enriched diet for 25 days. A third group of saline-injected offspring received the standard chow diet as a control. None of the saline-injected offspring was introduced to the NR diet. Twenty-five days later, social interaction was assayed using the three-chambered social interaction test [55].

Three-chambered social interaction test. To measure social behavior, the three-chambered social interaction test was performed as previously described [55]. The testing arena (model 60450, Stoelting Co., Wood Dale, IL, USA) had gray enclosures and plexiglass walls that divided the cage into three chambers. The chambers were connected via sliding plexiglass doors. During the first phase of the test, the experimental mouse was placed in the center chamber for 10 min, with the doors to the side chambers remaining closed. In the second phase of the test, the doors were opened, and the experimental mouse was allowed to explore all three chambers for 10 min. Then, the experimental mouse was returned to the center chamber, and wire cups were placed in each side chamber of the arena. One side had an empty cup, while the other had a cup with a novel mouse (the “target mouse”) that had been habituated to the cup in advance. During the third phase, the experimental mouse was allowed to explore all three chambers for 10 min. The time the experimental mouse spent sniffing the target mouse or an empty cup, as well as the time spent in each chamber, was quantified using the ANY-maze video-tracking system and software (version 7.4; Stoelting Co., Wood Dale, IL, USA). The preference index was calculated as a ratio of time sniffing a novel mouse to total sniffing time [56]. There was no difference between males and females; therefore, the data is presented without separating by sex.

The experimental unit in this experiment was a mouse (single animal). 12 mice were allocated to the MIA-control group, 11 mice to the MIA-NR group, and 10 mice to the Saline-control group. The total number of mice in the experiment was 33. No a priori sample size calculations were performed. All MIA mice available to us at PND143 were allocated to the MIA-control and MIA-NR groups, ensuring each group contained approximately equal numbers of males and females. A similar number of mice was allocated to the Saline-control group as those saline-treated on GD 12.5. No criteria were used to exclude the animals from the groups or time points during analysis; no exclusions were made. No confounders were controlled. No blinding was performed; experimenters were aware of the group allocation. An outcome measure for this experiment was the social preference index and time spent in the chamber with novel mice.

Statistical analyses. GraphPad Prism v9.3.1 was used to perform statistical analyses and generate graphs. All data are presented as mean ± SEM. The bioluminescent imaging data (prenatal treatment, DNP condition, n = 22–31/group) and social interaction time (treatment group x side, n = 10–12/group) were analyzed using a two-way ANOVA. The Fisher LSD test was used when main effects or interactions were observed. The social preference indices were compared using one-way ANOVA. Group differences were considered statistically significant when *p* < 0.05.

## 3. Results

To determine whether MIA alters mitochondrial function across development, the bioluminescent probe, MAL3, was used to measure ΔΨm in juvenile offspring, as previously described (Figure 1a) [45]. At PND22, measurement of basal ΔΨm level revealed no baseline differences in ΔΨm between MIA and control offspring (Figure 1c; Saline-MAL3: 5.44 ± 0.22 AU, MIA-MAL3: 5.95 ± 0.32 AU). To determine the ability of mitochondria to maintain ΔΨm under stress, one week later, mice received a low dose of DNP (1 mg/kg), a known uncoupler of oxidative phosphorylation, and ΔΨm was measured (Figure 1b). Control offspring showed no statistically significant difference in ΔΨm before and after DNP (Saline-MAL3 + DNP: 5.04 ± 0.14 AU). However, in MIA offspring, DNP produced a significant decrease (−15%) in ΔΨm-specific signal compared to baseline (MIA-MAL3 + DNP: 5.06 ± 0.18 AU). These results demonstrate that MIA impairs mitochondrial polarization maintenance under stress during the juvenile period, suggesting mitochondrial dysfunction.

To determine whether these mitochondrial deficits persist into adulthood, ΔΨm was measured in the same animals at PND 96 under baseline (Figure 1a) and DNP (Figure 1b) conditions. At PND96, measurement of basal ΔΨm level revealed no baseline differences in ΔΨm between MIA and control offspring (Figure 1d; Saline-MAL3: 5.45 ± 0.22 AU, MIA-MAL3: 5.65 ± 0.31 AU). Further, control offspring showed no significant change in ΔΨm before or after DNP injection (Saline-MAL3 + DNP: 5.19 ± 0.17 AU). However, in MIA offspring, DNP produced a significant decrease (−13%) in ΔΨm-specific signal compared to baseline (MIA-MAL3 + DNP: 4.92 ± 0.22 AU). Together, these results suggest that MIA produces persistent mitochondrial dysfunction in offspring.

**Figure 2:**
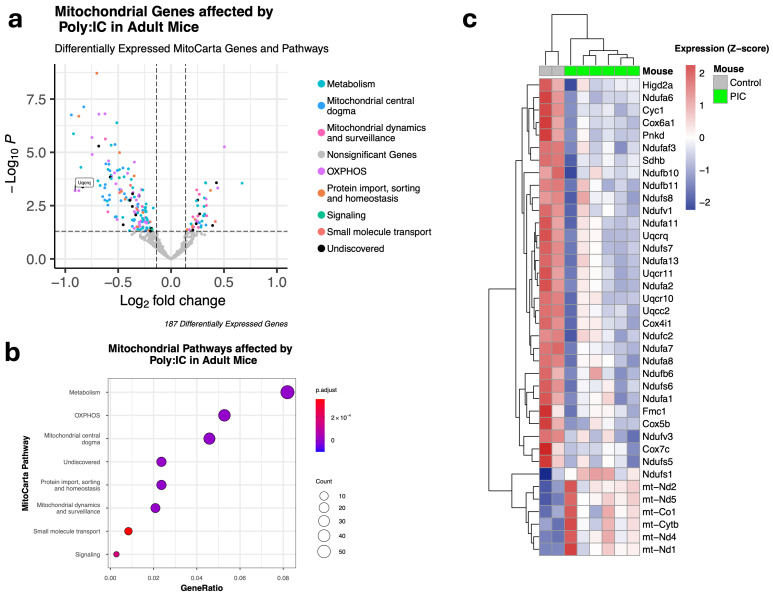
MIA alters the expression of genes associated with mitochondrial function. (**a**) MIA produced differential expression of 187 genes from the MitoCarta3.0 inventory. The corresponding MitoCarta3.0 pathway for each differentially expressed gene is illustrated. We observed 144 downregulated and 43 upregulated mitochondrial genes in the prefrontal cortex resulting from MIA. Differentially expressed OXPHOS gene, *Uqcrq*, which is also downregulated in patients with schizophrenia, is labeled. (**b**) Mitochondrial pathway analysis of differentially expressed mitochondrial genes using the MitoCarta3.0 dataset. GeneRatio and *p*-adjusted values indicate that genes primarily involved in metabolism and OXPHOS pathways were most prominently affected by MIA in the adult mouse prefrontal cortex. (**c**) Differentially expressed OXPHOS genes in the prefrontal cortex across all samples clustered by variance and colored by expression levels. n = 2 in the saline group and n = 6 in the MIA group.

To determine whether mitochondrial deficits are accompanied by changes in gene expression associated with mitochondrial function, we analyzed prefrontal cortex bulk RNA-seq data from a previous study as described above. Our differential expression analysis comparing control and MIA offspring identified 3724 differentially expressed genes, of which 187 were associated with mitochondrial function and included in the MitoCarta3.0 inventory (Figure 2a). Of these, 144 were downregulated, and 43 were upregulated. The differentially expressed MitoCarta 3.0 genes spanned all seven ‘MitoPathways,’ representing functional categories relevant to mitochondria. The MitoPathways with the most differentially expressed genes in MIA offspring were related to metabolism (N = 59) and OXPHOS (N = 38) (Figure 2b). The top 50 genes from the OXPHOS pathway with the most significant variance among samples after regularized log transformation are presented in Figure 2c.

**Figure 3:**
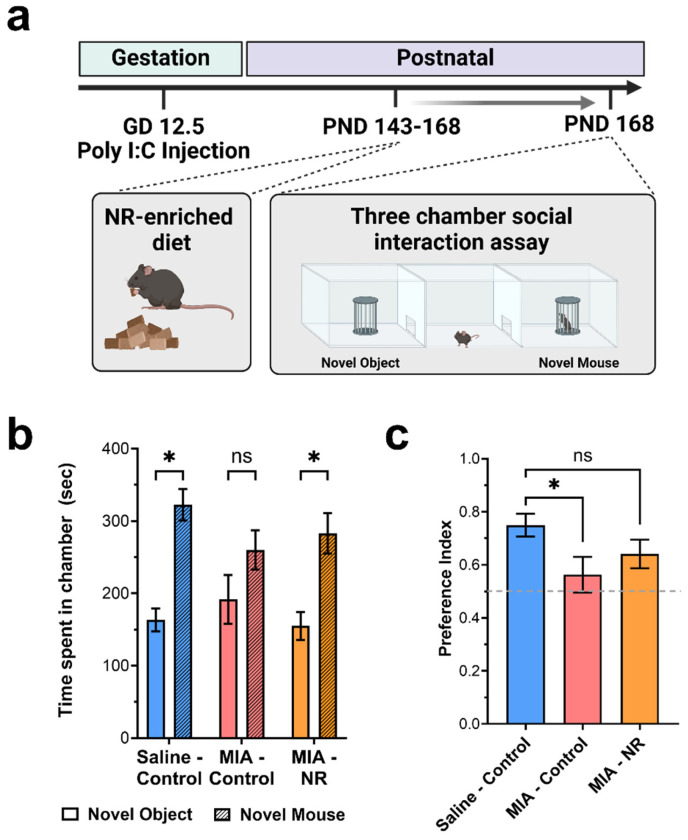
NR attenuates the MIA-induced deficit in social interaction. (**a**) Adult animals were fed the NR-enriched diet for 25 days, then tested on the 3-chamber social interaction test (Created in BioRender. Donegan, J. (2026) https://BioRender.com/b62mc6b). (**b**) Time spent in the chamber with a novel object or in the chamber with a novel mouse. (**c**) Normalized preference scores were calculated based on sniffing time. Results are presented as mean ± SEM. *p* values were calculated by two-way ANOVA (**b**) or one-way ANOVA (**c**); Fisher’s LSD test was used for the post hoc analysis. ns—*p* > 0.05, *—*p* < 0.05. n = 10 for the Saline—Control group, n = 12 in the MIA—Control group, and n = 11 in the MIA—NR group.

NR, the NAD^+^ precursor, is known to enhance mitochondrial function in various animal models [19,45,57]. We administered NR to MIA offspring for 25 days at a dose previously shown to be effective [58]. After this treatment, we measured social behavior using the three-chamber social interaction test [55,59,60] to assess whether mitochondrial deficits contribute to the behavioral outcomes associated with MIA (Figure 3a). As expected, control offspring demonstrated a preference for interacting with a novel mouse over a novel object. Specifically, control offspring fed the standard diet spent significantly more time in the chamber with the target mouse than in the chamber with the empty cup (Figure 3b; Saline-Control, Novel Object: 163.15 ± 15.85 s, Saline-Control, Novel Mouse: 322.31 ± 21.82 s) and showed a preference index > 0.5 (Figure 3c; Saline-Control: 0.75 ± 0.04). Conversely, MIA offspring fed the standard chow diet did not show a significant preference for interacting with the novel mouse over the novel object. Specifically, there was no significant difference in the time spent in the chamber with the novel object compared to the novel mouse (Figure 3b; MIA-Control, Novel Object: 191.6 ± 33.80 s; MIA-Control, Novel Mouse: 360.03 ± 27.02 s). Further, the preference index was significantly lower than that of control offspring (Figure 3c; MIA-Control: 0.56 ± 0.07). Administration of the NR diet restored social preference in MIA offspring (Figure 3b,c). Similar to control animals, MIA offspring fed the NR diet spent significantly more time in the chamber containing a novel mouse compared to the novel object (Figure 3b; MIA-NR, Novel Object: 154.76 ± 19.50 s; MIA-NR, Novel Mouse: 282.90 ± 28.03 s). Further, MIA offspring fed the NR diet demonstrated a social preference index that was not significantly different from control mice (Figure 3c; MIA-NR: 0.64 ± 0.05). Together, these results indicate that NR treatment attenuates the social interaction deficits caused by MIA.

## 4. Discussion

Activation of the maternal immune system during pregnancy is a known risk factor for the development of numerous neurodevelopmental and psychiatric disorders, including schizophrenia [10,11,61,62]. Prenatal influenza infection during the second trimester, for example, leads to an eight-fold risk of developing schizophrenia [10,11]. However, the specific pathogen matters less than the type of immune response [8,9]. Therefore, the viral mimetic polyinosinic/polycytidylic acid (Poly(I/C)), a double-stranded RNA that activates Toll-like receptor 3 (TLR3) and induces an acute Interferon Type I response in the dam and fetuses, is often used to model maternal immune activation (MIA) in rodents. Previously, high-molecular-weight Poly(I/C) has been shown to elicit a robust cytokine response in both maternal serum and fetal brain and to cause behavioral deficits in offspring [63,64]. It is important to note that MIA does not fully recapitulate all aspects of complex human disorders like schizophrenia; However, it is a useful model for understanding the neurodevelopmental processes that lead to neural circuit changes and deficits in complex behaviors later in life. Ex vivo studies demonstrate that MIA offspring exhibit altered mitochondrial gene and protein expression [41,42,43], reduced electron transport chain activity, increased oxidative stress, and reduced glutathione concentration [39,65], all indicative of mitochondrial dysfunction. Further, a decrease in ΔΨm was observed ex vivo in prefrontal cortical neurons isolated from MIA offspring [29,38].

While these results are intriguing, we provide the first in vivo evidence that MIA disrupts the maintenance of ΔΨm in both living juvenile and adult offspring in vivo in a non-invasive longitudinal manner. In the current experiments, we used the mitochondrial uncoupler, DNP, to demonstrate that MIA offspring lose the capacity to maintain ΔΨm under challenge. During uncoupling, ATP synthesis in mitochondria dissociates from proton pumping across the inner membrane [66], leading to changes in ΔΨm. Our experiments, to our knowledge, provide the first evidence that MIA impairs the capacity to maintain ΔΨm during development. This finding is especially important, as mitochondria have been implicated in key neurodevelopmental processes (e.g., axon and dendrite growth [67,68,69,70], synaptogenesis [36,71,72], synaptic pruning [36]). Although the MAL3 provides information on mitochondrial polarization over time in vivo, it does not allow for defining the metabolic profile of cells and tissues involved in the development of behavioral deficits, e.g., the prefrontal cortex. Our future work will focus on measuring electron transport chain activity and oxygen consumption in prefrontal cortical neurons.

A recent report demonstrated that the transfer of healthy mitochondria to MIA offspring alleviated mitochondrial dysfunction and deficits in spontaneous locomotor activity [27]. These results align with our current findings, which demonstrate that treatment with NR, a form of vitamin B3 that we have shown to increase ΔΨm [45], attenuated social interaction deficits in MIA offspring. Interestingly, there was no significant difference between males and females in the social interaction. Therefore, the data is presented without being separated by sex. NR elevates intracellular NAD^+^ levels and exerts multiple positive effects on energy metabolism and neuroprotection [73,74]. It has been shown that restoring the NAD^+^ pool in mammalian cells, *C. elegans*, and mice with dysfunctional mitochondria induces mitophagy and mitobiogenesis [57,58], thereby replacing damaged and dysfunctional mitochondria with functional, bioenergetically active ones. While it is likely that the effect of NR on social behavior resulted from improved mitochondrial function, the exact mechanism remains unclear, including the brain regions and neurotransmitters involved. A recent study by Gerasimenko et al. found that NR supplementation increased social preference in CD157 KO mice [75]. CD157 is a cell surface molecule that, along with CD38, converts NAD^+^ to cyclic ADP-ribose, a second messenger that mobilizes calcium. In the hypothalamus, cyclic ADP-ribose increases intracellular Ca^2+^, leading to oxytocin release. The authors demonstrate that in CD157 KO mice, NR increased oxytocin production and release, a hypothalamic hormone that plays an important role in social behaviors [76]. While NR treatment may have increased oxytocin levels in MIA offspring, no changes in oxytocin levels were observed in wild-type (C57BL/6N) mice treated with NR, suggesting that other neural circuits may be involved. Lastly, a recent report also indicates increased NADH- and succinate-linked mitochondrial respiration, as well as maximal electron transfer capacity, in the prefrontal cortex and amygdala of adult MIA offspring, suggesting a compensatory response after stress induced by Poly(I/C) [40]. While the current study does not provide a mechanistic link between NR uptake and improvement in socialization, future experiments will examine the effects of NR on the prefrontal cortex, a brain region implicated in social behavior [77]. Specifically, we will monitor changes in gene expression, protein levels, NAD+ levels, and the capacity to maintain ΔΨm, selectively in the prefrontal cortex and in neurons isolated from it.

Further, we analyzed a previously collected and publicly available dataset in which bulk RNA-Seq was performed on prefrontal cortex tissue from MIA or control offspring. The goal of this targeted re-analysis was to determine whether MIA produces large-scale changes in mitochondrial gene expression and to identify genes and pathways for future investigation. Our analysis, which used the MitoCarta3.0 inventory to focus solely on mitochondrial genes, indicated persistent alterations in the expression of genes associated with mitochondrial function. All seven functional mitochondrial pathways were affected. In line with the observed changes in ΔΨm, the pathways with the most differentially affected genes included metabolic processes and oxidative phosphorylation (OXPHOS). Among these differentially expressed mitochondrial genes, one of the largest fold changes was observed in the OXPHOS gene *ubiquinol-cytochrome c reductase complex III subunit VII* (*Uqcrq*). *Uqcrq* is primarily located in the mitochondrial inner membrane, where it participates in the mitochondrial respiratory chain, transferring electrons from ubiquinol to cytochrome c during OXPHOS. We found that *Uqcrq* was significantly downregulated in adult MIA offspring, consistent with post-mortem tissue from schizophrenia patients. Specifically, *Uqcrq* expression is significantly decreased in dorsolateral prefrontal cortex pyramidal cells from schizophrenia patients compared to healthy controls [19,78]. Overall, these results indicate that prenatal exposure to MIA in mice significantly affects the expression of mitochondrial genes in the mature prefrontal cortex. This finding is consistent with a previous report on the effect of MIA on the expression of genes that alter mitochondrial function [40]. Although the observed effects of MIA on gene expression were replicated across independent cohorts and experimental settings, these findings collectively strengthen confidence in the robustness and reproducibility of the results. Nevertheless, future studies are needed to address current limitations by increasing sample size, confirming gene and protein expression changes in the same cohorts used for behavioral testing, and elucidating the functional relevance of these molecular alterations to behavioral outcomes.

## 5. Conclusions

Schizophrenia is a heterogeneous psychiatric disorder that includes positive symptoms (e.g., hallucinations and delusions), negative symptoms (e.g., anhedonia and social withdrawal), and cognitive symptoms (e.g., working memory deficits, attentional issues) [79]. While currently available antipsychotics are relatively effective at treating positive symptoms of the disorder, negative and cognitive symptoms are often left untreated [80]. These symptom domains often have the greatest impact on day-to-day functioning and disease outcomes [81,82]. Therefore, there is an urgent need to identify new treatment strategies to improve all symptoms of schizophrenia. In the current experiments, we present initial evidence that NR alleviates social interaction deficits in MIA offspring. Future experiments will build on these findings by defining the specific mitochondrial mechanisms by which MIA impairs neurodevelopment and produces behavioral deficits. Further, we will determine the translational relevance of our results to schizophrenia pathology, with the ultimate goal of identifying novel treatment strategies for schizophrenia. In conclusion, the current studies provide the first in vivo evidence of altered ΔΨm in MIA offspring, and future experiments will be designed to better understand the cellular mechanisms by which this contributes to neural circuit alterations and behavioral deficits

## Data Availability

Data are provided in the manuscript, and the raw files are available from the authors upon request.

## Abbreviations

The following abbreviations are used in this manuscript:

GD	Gestational day
PND	Post natal day
NAD^+^	Nicotinamide adenine dinucleotide
NADH	Reduced nicotinamide adenine dinucleotide
